# Socio-economic and psychological factors contributing to drug use among females in Khyber Pakhtunkhwa, Pakistan: A mixed-methods approach

**DOI:** 10.4102/sajpsychiatry.v32i0.2506

**Published:** 2026-01-21

**Authors:** Muhammad Suhail Khan, Wu Zongyou, Abdur Rahman, Aman Khan, Shagufta Batool

**Affiliations:** 1 Department of Sociology, School of Sociology and Political Science, Anhui University, Hefei, China; 2 Department of Psychology, International Islamic University Islamabad, Islamabad, Pakistan; 3 Department of Sociology, School of Public Administration, Hohai University, Jiangsu, China; 4 School of Psychology, Northeast Normal University, Changchun, China

**Keywords:** female drug use, socio-economic determinants, psychological stress, cultural stigma, gender-sensitive interventions

## Abstract

**Background:**

Women in Khyber Pakhtunkhwa face several barriers, including poverty, cultural stigma, intense social pressure and a lack of accessible services, which prevent them from addressing drug use and mental health issues.

**Aim:**

The study aimed to explore the impact of socio-economic and psychological factors on substance use among women aged 25–34.

**Setting:**

This study was conducted at community centres and private schools across several cities in Khyber Pakhtunkhwa, Pakistan, including Peshawar, Charsadda, Nowshera, Swabi and Mardan.

**Methods:**

A mixed-methods approach was employed, combining quantitative surveys from 120 participants to analyse drug use patterns and qualitative interviews with 20 participants to investigate personal and social challenges through in-depth interviews.

**Results:**

Quantitative data indicated that unemployment, financial hardship and low education significantly predicted drug use, whereas moderate depression, anxiety and stress exacerbated it. Perceived stigma serves as a protective role in this study. Qualitative findings highlight themes such as socioeconomic challenges, mental health issues, cultural pressures and barriers to treatment that exacerbate these problems.

**Conclusion:**

This study highlights the importance of rehabilitation centres and community counselling services tailored to women’s specific needs, along with legislative measures to address gaps in support and resources for women facing drug use issues.

**Contribution:**

This study contributes to the understanding of the complex relationships between mental health, socioeconomic status and cultural factors influencing drug use among women and aims to inform public health policy and strategy.

## Introduction

The challenge of drug use among young women has emerged as a critical global concern, carrying profound social, economic and health implications.^1^ Substance dependence, once predominantly an issue among men, is increasingly impacting young women, particularly as women resort to drugs to manage stress, depression and financial hardship.^2^ Studies on drug use primarily focus on men’s perspectives, sparing little attention for young women.^3,4^ Despite the growing global awareness of the seriousness of drug problems, traditional societies such as Khyber Pakhtunkhwa continue to receive little attention.^5^ Women in KP encounter distinct obstacles, such as constrained access to psychiatric services and limitations on mobility stemming from cultural stigma.^6^ Elevated unemployment levels, diminished literacy rates and socio-economic challenges frequently render women susceptible to substance use.^7^ The increase in drug use in KP is particularly concerning, especially among young girls under the age of 25 years.^8^ Despite KP being a traditionally male-dominated society, the rise in drug use among women in the area is becoming more prevalent, presenting a considerable public health challenge.^9^ Socio-economic challenges like poverty, restricted education and family dynamics frequently drive young women to resort to drug use as a means of coping with trauma, anxiety and depression.^10^

Social and cultural norms, along with stigma, hinder mental health treatment and worsen mental health issues, especially for women.^11^ Women frequently endure psychological suffering in silence to protect family reputation, resulting in isolation and, in some instances, substance use.^12^ Substance use among women is increasing rapidly, reflecting changes in social norms and various mental and economic factors.^13^ Historically, men have had higher rates of substance use.^14^ Recent studies show a narrowing gender gap in developed countries, with women’s drug use approaching men’s, including increased alcohol, prescription medications and illegal drugs, because of biological and psychological differences.^15^ In developing regions, cultural barriers prevent open discussions about substance use, leading women to use drugs as a coping mechanism for issues like income insecurity and social injustice.^16^ Studies reveal under-reporting of women’s drug use in South Asia, despite societal prohibitions, and policy discussions often overlook the growing issue of female substance use and the need for support and dignity.^17,18^

Recent research on women’s drug usage reveals the complexity of female addiction, often misunderstood as a coping mechanism for mental health issues, emotional distress and social pressures.^19^ Women, particularly those with a drug history, frequently rely on prescription medications to manage conditions like post-traumatic stress disorder and anxiety because of loneliness, family challenges and relationship issues.^20^ Additionally, hormonal and metabolic differences may increase women’s vulnerability to addiction and health risks.^19,20^ Research on female drug use is emerging in South Asian countries like Bangladesh and India but remains limited and primarily focused on urban areas.^21^ Many women hide their substance use because of stigma and gender bias, as noted in a qualitative study from Afghanistan.^22^ Despite increasing public health awareness of women as drug users, many countries lack gender-sensitive treatment options and research on female drug use, especially in traditional settings and countries with limited services and cultural barriers, like Pakistan.^23^

The literature on drug consumption patterns in KP Province, Pakistan, reveals significant gender gaps in studies on drug use, despite the pressing public health issue being primarily studied in men or urban populations.^24^ The societal norms and regulations in this region restrict women’s access to resources and support, obscuring the environment surrounding female drug addiction.^25^ The overlapping routes of drug trafficking in KP have exacerbated the situation, leading to an increase in drug supply.^26^ In remote rural areas, the accessibility of drugs is on the rise.^27^ Young women often encounter drugs, which can foster connections or help alleviate life’s pressures, through their peers in academic settings.^28^ Social networks have contributed to the normalisation of drug use among young women, prompting them to experiment with more affordable synthetic drugs.^29^ This trend highlights the fact that the absence of suitable rehabilitation facilities that consider gender-specific needs does not address the distinct requirements of female addicts.^30^

Rehabilitation programmes frequently focus on men, overlooking the unique support that women require to address drug addiction effectively.^31^ Limited research on Pakistani women’s drug use reveals psychological and economic factors such as financial hardships, dependence on male relatives and barriers to education and employment.^32,33^ The literature on drug use in Pakistan is inadequate, and the psychosocial factors linked to drug use are poorly understood, leading many women to resort to drugs as a coping mechanism.^10,34^ This study explored the socio-economic and psychological factors affecting drug use among women in KP and analysed the interplay between these elements and cultural and familial stressors. Our study seeks to address the following research questions: (1) What are the socio-economic and psychological factors contributing to drug use among women in KP? (2) How do cultural norms and family dynamics influence female drug use in this region?

## Research methods and design

### Research design

We used a sequential mixed-methods explanatory design. The quantitative phase uses surveys to discover broad tendencies, while the qualitative phase uses semi-structured interviews to understand drug experiences. We conducted surveys and used quantitative approaches to uncover psychological, social and cultural elements that cause drug addiction in women. We measured psychological distress, including depression, anxiety and stress, using the Depression, Anxiety and Stress Scale 21 (DASS-21), while socio-economic distress, including income, unemployment and family financial pressures, was assessed using tailored survey items. We collected the data from February to December 2022. The research included 120 participants, aged 25–34 years, who were female residents of the cities of Peshawar, Charsadda, Nowshera, Swabi and Mardan in KP Province, Pakistan. Firstly, we chose this demographic because they may be sensitive to socio-economic and psychological variables that drive drug use. Secondly, we considered the shared socio-economic obligations, jobs or family stresses and mental illnesses among 25–34-year-olds. Financial, societal and familial factors may cause drug use in this age group. The qualitative step involves follow-up interviews to provide context and quantitative results, combining both methodologies to provide a comprehensive understanding of personal and cultural circumstances. This mixed-methods approach captures patterns and personal tales, deepening comprehension.

### Phase 1: Quantitative phase – Survey

*Data Collection Instrument*: We used the DASS-21 scale to measure mental health in a sample of 120 female participants. The scale was used to assess psychological distress, including depression, anxiety and stress levels and socio-economic factors like financial stress, unemployment and family financial pressures through a tailored survey. The items were assessed using a Likert scale, ranging from 1 (strongly disagree) to 5 (strongly agree). The DASS-21 scale and additional survey items were adapted to the socio-economic and cultural context of women in KP, Pakistan, for community validation. Female research assistants and snowball sampling were employed to build trust and overcome cultural barriers, ensuring the instrument’s relevance and acceptance in the local community.

#### Sample size and participant recruitment

We determined a sample size of 120 women who use drugs in KP Province, Pakistan, based on the study’s objectives, the available resources and the need to represent the population comprehensively. We recruited 120 participants for quantitative methods to identify patterns and understand the socio-economic and psychological factors influencing drug use among women in the region. The sample size was chosen because of the sensitive nature of the topic and the logistical challenges of recruiting women participants, ensuring diversity of perspectives and feasibly collecting data for the study. We utilised healthcare providers and social workers to recruit participants from local networks, using a snowball sampling method. This method helped build trust in the community by engaging participants who were familiar with drug use and related issues. Because cultural norms in the region limit men’s interactions with women, we hired female research assistants to conduct the surveys, which allowed us to overcome social barriers and ensure that participants felt comfortable discussing sensitive topics such as drug use and mental health. Using female research assistants helped build rapport with participants, encouraging open and honest discussions about the participants’ experiences.

#### Data analysis

The analysis employed both descriptive and inferential statistical techniques to evaluate quantitative data collected from 120 participants. In the initial step, we analysed the demographic data of the participants, including age, marital status, education level, employment status and income. We utilised frequency distributions and percentages for categorical variables, such as marital status, and mean values for continuous variables, including age and monthly income. Descriptive statistical methods were used to summarise the data, including the frequency of drug use, while inferential statistics were employed to explore associations among psychological traits, socio-economic factors and drug-use behaviours. The strength and direction of the relationships among psychological factors, socio-economic factors, social influences and drug-use behaviours were assessed using Pearson’s correlation coefficient. The analysis through multiple regression revealed the key predictors of drug use, which encompass factors such as depression, anxiety, stress, income and employment status, family support, stigma and cultural expectations. The frequency of substance usage served as the dependent variable. Standardised beta coefficients (*β*) and *p*-values were utilised to assess the impact of each predictor. The survey results were processed using SPSS, employing mean imputation for continuous variables and mode imputation for categorical data. We used the normality, linearity and multicollinearity assumptions of regression analysis to verify the accuracy of the statistical models.

### Phase 2: Qualitative phase – Semi-structured interviews

*Data Collection Method:* The investigation included semi-structured interviews with 20 participants who consented to engage further. The interviews happened in secure and comfortable environments such as community centres and private schools, concentrating on individual experiences related to substance use, mental health issues, family dynamics, cultural stigma, social pressures and obstacles to accessing treatment and support.

#### The interview process and procedure

This investigation employed interviews to analyse participants’ experiences with drug use in the cities of Peshawar, Charsadda, Nowshera, Swabi and Mardan in KP Province, Pakistan. With the help of healthcare providers and social workers, we identified participants who use substances by collaborating with local networks of women who have direct knowledge of drug use in the community. The interviews were categorised into four distinct sections: general demographic information, social influences contributing to drug use among women, psychological issues linked to drug use and economic factors. The data were carefully documented, ensuring a precise translation to uphold quality standards. Clear and straightforward questions were employed to gauge participants’ perspectives. Clarifications were offered to enhance comprehension of the underlying factors contributing to drug addiction. We conducted interviews over 3 h, ensuring that participants consented to documentation. The team assured participants that their information would be kept confidential. The investigation seeks to deliver an in-depth analysis of substance dependence and the multiple factors contributing to the dependence.

#### Sample size and sampling technique

A total of 20 participants were chosen from a pool of 120 in the quantitative survey to capture a range of personal and cultural experiences associated with drug use. The qualitative phase sought to investigate the socio-economic, psychological and cultural influences on drug use, integrating the lived experiences and diverse backgrounds of participants. The participants involved demonstrated a willingness to proceed with the interview process, offering significant insights that enriched the study results.

#### Data analysis – Thematic analysis

A thematic analysis of the interview transcripts was conducted using NVivo software to explore the qualitative data through an iterative process. Transcripts were initially coded by identifying recurring concepts and phrases that appeared across multiple interviews. We carefully reviewed the initial codes to ensure their consistency and accuracy in reflecting participants’ experiences. The initial round of coding revealed several key themes, including financial hardship, emotional strain, social stigma and family pressures. We analysed each theme to understand the factors influencing substance use. The main themes that emerged from our initial categorisation include socio-economic challenges, mental health and emotional struggles, cultural and familial pressures and barriers to treatment and rehabilitation. We compared and contrasted themes to deepen our understanding of how socio-economic and cultural factors influence drug use among women in KP. By analysing both data sets, the study confirmed the overlapping findings and identified unique nuanced points that the survey alone might not have captured.

### Ethical considerations

Ethical clearance to conduct this study was obtained from the Ethics committee of Anhui University (No: LX202302149). Interviewed participants provided written informed consent with the signatures of two witnesses.

## Results

The findings section presented quantitative and qualitative findings, providing a comprehensive understanding of socio-economic and psychological factors influencing drug abuse among women in Pakistan.

### Quantitative results

Table 1 presents a detailed analysis of 120 female participants aged 25 years to 34 years, with demographic and socio-economic characteristics. The most represented age groups were 30 (14.1%) and 33 (13.3%), with a notable focus on young to early middle-aged women facing various life transitions. The majority, 65.8%, were single, while 19.1% were divorced, indicating diverse marital statuses that may influence their socio-economic and psychological experiences. Regarding education, 30% of participants obtained a master’s degree or higher, while 24.1% held a bachelor’s diploma. However, 17.5% lacked formal education, and smaller percentages have completed primary (5.8%), secondary (10%) or higher secondary education (12.5%). This statistic highlights a significant disparity in educational attainment among the participants. The unemployment rate was notably high, with 70% of participants jobless. Only 11.6% were employed, and 18.3% were self-employed, reflecting financial vulnerabilities, especially when job opportunities for women are limited. Furthermore, 75.8% of participants report severe financial difficulties, earning less than 5000 PKR monthly, with only a tiny fraction earning higher amounts. This financial hardship exacerbates the challenges faced by these women.

**TABLE 1:** Demographic and socio-economic profile (*n* = 120).

Characteristics	*N*	%
**Gender**		
Women	120	100.0
**Age (years)**		
25	09	7.5
26	13	10.8
27	15	12.5
28	07	5.8
29	14	11.6
30	17	14.1
31	07	5.8
32	12	10.0
33	16	13.3
34	10	8.3
**Marital status**		
Single	79	65.8
Married	09	7.5
Divorced	23	19.1
Widowed	09	7.5
**Education level**		
No formal education	21	17.5
Primary	07	5.8
Secondary	12	10.0
Higher secondary	15	12.5
Bachelor’s degree	29	24.1
Master’s degree or higher	36	30.0
**Employment status**		
Unemployed	84	70.0
Employed	14	11.6
Self-employed	22	18.3
**Monthly income (PKR)**		
< 5000	91	75.8
11 000–15 000	11	9.1
16 000–20 000	13	10.8
21 000–25 000	05	4.1
> 25 000	0	0.0

PKR, Pakistan Rupee.

**FIGURE 1:**
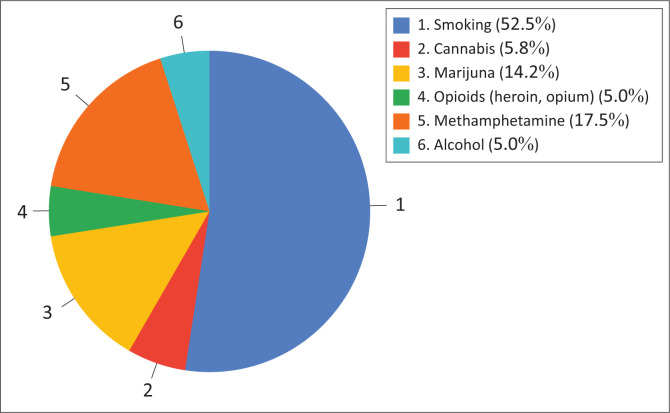
Proportion of substance use by type.

The pie chart (Figure 1) illustrates the distribution of substance use among participants, highlighting the prevalence of various substances in the study group. Smoking, which constitutes 52.5% of all cases, accounted for the most significant proportion, highlighting the prevalence and routine use of smoking among participants. Methamphetamine (‘ice’) came in second at 17.5%, indicating its prevalence as a substance of occasional-to-moderate use. Cannabis was the third-most prevalent substance, accounting for 14.2% of use, primarily associated with recreational activities. Of the participants, 5.8% said that they used cannabis, highlighting its importance in social and recreational settings. Alcohol and opioids (heroin and opiates) each accounted for 5.0% of total use, indicating a clear but limited trend in consumption. The study reveals that smoking is the leading substance-use behaviour, with methamphetamine and marijuana also prevalent. Other substances such as marijuana, alcohol and opioids account for a smaller but significant proportion. These findings highlight the importance of managing stress and anxiety effectively.

**FIGURE 2:**
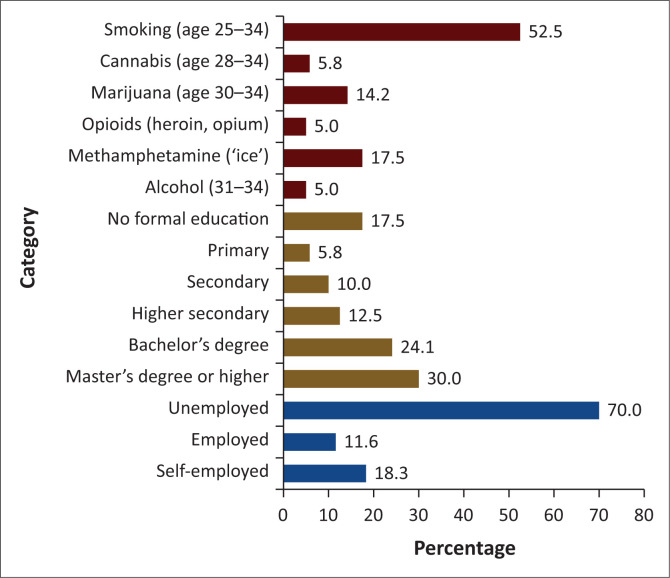
Substance use patterns by age, education and employment status.

Figure 2 illustrates the Substance Use Patterns categorized by Age, Education, and Employment Status, revealing that 25–34-year-olds smoked 52.5%, followed by methamphetamine (‘ice’) and marijuana at 17.5% and 14.2%. Only 5.8% of 28–34-year-olds use cannabis, and 5% use opioids. Alcohol use is low among 31–34-year-olds at 5%. Substance usage prevalence is most significant among those with a master’s degree or above 30%, followed by bachelor’s degree holders at 24.1%. Those with less education report far lower percentages, with 17.5% having no formal education, 12.5% having upper secondary education and 5.8% and 10% having elementary and secondary education. This evidence shows that highly educated people utilise stress-related substances more efficiently. Drug usage is 70% among jobless people, 18.3% among self-employed people and 11.6% among employed people. Economic instability and unemployment increase stress, worry and lack of structure, leading to drug use as a coping strategy.

### Descriptive statistics for mental health variables

Table 2 presents the descriptive data related to mental health. We used descriptive statistics to summarise the DASS-21 scores of participants related to depression, anxiety and stress. The data and standard deviation indicate the average psychological suffering within the sample. We implemented this approach by calculating an average of seven items from each subscale of DASS-21: depression, anxiety and stress. Participants evaluated each question using a 4-point Likert scale: (0: Did not apply to me at all; 1: Applied to me to some degree or some of the time; 2: Applied to me to a considerable degree or a good part of the time and 3: Applied to me very much or usually). The test scores indicate the severity of symptoms associated with mental disorders, with significant patterns and variability in responses. The mean scores highlight mental health factors, while the standard deviation reflects individual responses.

**TABLE 2:** Descriptive statistics for mental health variables (*n* = 120).

Mental health variable	Mean	s.d.
Depression	1.48	1.11
Anxiety	1.41	1.12
Stress	1.50	1.11

s.d., standard deviation.

We found a moderate variation in the scores, *M* = 1.48 and standard deviation (s.d.) = 1.11. The participants’ average depression levels were moderate, with some showing mild symptoms and others showing more pronounced levels. We observed moderate anxiety symptoms, resulting in an *M* = 1.41 and an s.d. = 1.12 between anxiety and depression. This value indicates that anxiety scores exhibited a moderate level of variability as well. We observed more evident stress symptoms, leading to *M* = 1.50. The variability in stress levels aligned with that of depression and anxiety, exhibiting an s.d. of 1.11. This value indicates that the psychological distress reported by the sample stays the same. For depression, anxiety and stress, most of the respondents scored within one standard deviation of the mean, which indicates that they have a thorough understanding of their mental health.

### Correlation analysis between mental health and drug-use frequency

Table 3 shows the correlation analysis between mental health indicators and drug-use frequency. We used statistical tests to analyse the correlation between mental health variables and drug-use frequencies, aiming to determine whether there was a significant relationship. Mental health was assessed using the DASS-21, which consists of three subscales (depression, anxiety and stress), each containing seven items rated on a 4-point Likert scale. Participants’ responses were summed to create the total scores for each mental health indicator. Drug use was quantified through a ‘drug use percentage’, providing a precise continuous measure of each subject’s frequency of drug consumption. The Pearson correlation coefficient was utilised to assess the strength and direction of the linear relationship between mental health indicators and drug use. This method helps to determine how changes in one variable may influence changes in another, indicating a polynomial relationship. The coefficient, denoted as R, ranges from −1 to +1: A value above +1 indicates a perfect positive correlation, a value below −1 indicates a perfect negative correlation and 0 indicates no correlation.

**TABLE 3:** Correlation analysis between mental health and drug use.

Mental health indicator	Correlation coefficient (*r*)	*p*-value
Depression	−0.14	0.12
Anxiety	−0.09	0.32
Stress	0.04	0.66

We conducted a statistical hypothesis test to examine the correlation between mental health scores (depression, anxiety and stress) and medication-use frequency, using a significance level of 0.05. The null hypothesis (H2) stated that there was no correlation (*r* = 0), while the alternative hypothesis (H1) suggested that a significant correlation (*r* ≠ 0) existed. The results showed a *p*-value below 0.05, indicating a significant linear relationship, which led to the rejection of the null hypothesis. Conversely, a *p*-value above 0.05 would suggest that any observed correlation is likely as a result of chance. The accompanying table presents the correlation coefficients and *p*-values.

The correlation analysis between mental health and drug use yielded an *r* = −0.14 and *p* = 0.12. The values indicate a weak and non-significant correlation between higher levels of depression and a slight decrease in drug use among participants. The *p*-value is more significant than *p* > 0.05, indicating that the observed connection could be coincidental rather than an actual causal link. Self-anxiety scores showed no significant association with *r* = −0.09 and *p* = 0.32. The scores indicate a low-to-moderate negative correlation between anxiety levels and drug-use frequency, suggesting that individuals with higher anxiety may use drugs less often. However, the correlation was not statistically significant (*p* > 0.05), meaning that drug-use frequency does not reliably predict anxiety levels. The correlation involving the stress score was weak, with *r* = 0.04 and *p* = 0.66. The coefficient indicates that stress does not significantly impact the regularity of drug use, despite its low and positive mobility. The obtained *p*-value being more significant than *p* > 0.05 indicates that these relationships are not statistically significant.

### Socio-cultural and family influences on substance use

Table 4 shows the responses from a Likert scale about stigma, family support, mental health resources and the critical connections among family relationships, societal pressures and drug use. We used three distinct categories to organise the Likert-scale items into which participants rated their responses. (1). Family support encompasses emotional and practical assistance from relatives and the community. (2). The stigma and social perception encompass judgement and societal expectations. (3). The accessibility of resources encompasses the awareness, availability and adequacy of mental health services. We recorded the responses using a 5-point scale: (1 = strongly disagree, 2 = disagree, 3 = neutral, 4 = agree and 5 = strongly agree). We used descriptive statistics to calculate the means (*M*), s.d. and agreement percentages.

**TABLE 4:** Descriptive statistics on family support, stigma and resource access.

Category	Statement	Mean	s.d.	% Agree or strongly agree
Family support	My family provides emotional support when I am struggling.	2.36	7.53	47.5
	My community offers support for individuals facing substance-use issues.	1.61	8.00	32.5
	I can rely on family or friends if I need help dealing with personal problems.	2.00	2.82	40.8
Stigma and social perception	I feel judged by others in my community because of my personal choices.	2.30	6.78	50.0
	People in my community look down on women who face substance-use issues.	2.71	12.42	63.3
	Cultural expectations place pressure on me to behave in a certain way.	2.28	4.81	48.3
	I feel that my family or community understands my challenges.	1.58	7.53	26.6
Access to resources	I am aware of the available mental health services in my area.	2.30	7.45	50.0
	I have access to resources that can help me with mental health challenges.	2.25	9.67	39.1
	Seeking help for substance use is generally accepted within my community.	2.50	9.38	56.6

s.d., standard deviation.

The findings indicate that only 40.8% of participants could rely on family or friends for emotional support during personal challenges, while 47.5% acknowledged this finding. Of the participants, 32.5% reported drug-related issues, with many feelings constantly monitored. Cultural expectations significantly influenced stress, with 26.6% reporting understanding from family or community. Only 39.1% felt restricted in accessing mental health services despite half being aware of their availability (see Table 4).

The correlation analysis revealed that family emotional support was significantly negatively correlated with substance use (*r* = −0.28, *p* = 0.01), indicating that higher support is associated with lower substance use. The study found a significant positive correlation between perceived stigma and substance use (*r* = 0.30, *p* = 0.01), indicating that a higher level of stigma increases the likelihood of substance use. Cultural expectations (*r* = 0.22, *p* = 0.08) and awareness of mental health resources (*r* = −0.12, *p* = 0.18) showed non-significant correlations (see Table 5).

**TABLE 5:** Significant correlation analysis.

Variable pair	Correlation coefficient (*r*)	*p*-value	Significance
Family emotional support versus substance use	−0.28	0.01	Significant
Perceived judgement (stigma) versus substance use	0.30	0.01	Significant
Cultural expectations versus substance use	0.22	0.08	Not significant
Awareness of mental health resources versus substance use	−0.12	0.18	Not significant

Regression analysis indicated that emotional support and perceived stigma from family were significant predictors of the frequency of substance use. Increased family emotional support (*β* = −0.25, *p* = 0.02) was a protective factor, reducing the likelihood of substance use and underscoring the importance of interventions focusing on family dynamics. In contrast, perceived stigma (*β* = 0.28, *p* = 0.01) was associated with increased substance use, suggesting that social judgements exacerbated stress and discouraged seeking help. Analysis indicated that cultural expectations and awareness of mental health resources were not statistically significant predictors (*p* = 0.10 and *p* = 0.21). This finding suggests that they may influence behaviour but not significantly (see Table 6).

**TABLE 6:** Regression analysis: Predictors of substance-use frequency.

Predictor variable	Standardised coefficient (*β*)	*p*-value	Significance
Family emotional support	−0.25	0.02	Significant
Perceived judgement (stigma)	0.28	0.01	Significant
Cultural expectations	0.18	0.10	Not significant
Awareness of mental health resources	−0.12	0.21	Not significant

The influence of family support, social stigma and resource availability on drug use is strongly supported by quantitative evidence. Moderate emotional support from family was observed in 47.5% of individuals, serving as a deterrent against drug use. Stigma, mainly regarding women, affected 63.3% of the participants. The correlation between stigma and drug use highlights the critical need for educational initiatives that are both anti-stigma and culturally sensitive. While 50% of the participants were aware of mental health services, merely 39.1% reported having sufficient access, indicating that awareness alone does not ensure availability. Psychological elements such as familial support and societal stigma were found to be more predictive of drug use than economic considerations (see Table 4).

### Qualitative results

Qualitative data interviews revealed personal accounts of substance use, highlighting socio-economic, mental health and cultural challenges, emphasising their profound impact on participants’ lives.

### Socio-economic challenges

‘The organisation I worked for closed, resulting in unemployment for many, including myself. The scarcity of job opportunities and skills has led to a decline in my free time and savings. To cope with the anxiety and stress from financial instability, I, along with some friends, have turned to substance use.’ (Participant-3, Age 32, Gender Female)

‘The father’s illness caused financial strain, leading me to drop out of school and take on low-paying jobs. This exhaustion drove me to seek relief through drug use as a way to cope with the stress of my situation.’ (Participant-9, Age 29, Gender Female)

‘I face financial challenges as a single parent with three children and a husband earning minimum wage. I initially used prescription painkillers for physical and mental relief.’ (Participant-17, Age 34, Gender Female)

The narratives highlight the adverse effects of economic instability on women, particularly their substance use because of unemployment, financial difficulties and wealth inequality, which in turn leads to anxiety and substance use. Women in poverty often face low-quality employment, leading to psychological distress and drug use.^35^ Married adults utilise prescription medications for both physical and psychological relief, as managing finances and raising children in poverty pose significant emotional risks.^36^

### Mental health and emotional struggles

‘I struggle with anxiety due to various factors and am unable to afford expensive medications. I use cheap drugs as a temporary solution to alleviate constant panic and stop mind chatter.’ (Participant-2, Age 34, Gender Female)

‘My family’s incomprehension deepened my sadness. I turned to drugs for comfort because I felt estranged. Despite the physical and mental devastation, my only companion is my Kindle, which I value.’ (Participant-19, Age 29, Gender Female)

‘Grief at my brother’s abrupt death made me physically sad. I resorted to drugs to fill the emptiness left by his death when conventional support failed. That is my only relief since it temporarily breaks the mourning cycle.’ (Participant-11, Age 33, Gender Female)

The narratives highlight the significant mental health challenges that drive women to resort to narcotics as self-treatment. Financial constraints and social isolation exacerbate this distress, leading to over-the-counter medications.^37^ The profound pain caused by unaddressed grief, which is often unbridled by existing support systems. Factors such as restricted access to mental health services, family communication breakdowns and emotional distress contribute to female drug misuse.^38^

### Cultural and familial pressures

‘I face prejudice from family and friends as a 27-year-old single person. Even though I may be judged at social events, I use drugs to cope with the social stigma and pressures that value me based on outdated standards.’ (Participant-5, Age 27, Gender Female)

‘My life changed when my parents planned my marriage to a stranger, over which I had no influence. This marriage has been miserable and lonely, so I turned to drugs to escape it and the feeling that my decisions were being made for me.’ (Participant-18, Age 27, Gender Female)

‘My society still shames divorce, particularly for women. People in my neighbourhood saw me differently after my divorce. They considered me exploited. Removing myself from a community where I’m not judged and excluded, even from my family, is difficult. Despite my loneliness, I used drug addiction to cope with scorn and societal rejection.’ (Participant-20, Age 34, Gender Female)

The narratives highlight the significant impact of cultural and family pressures on drug abuse among women in the region. The narratives emphasise the role of social stigma, forced unions and the exclusion of divorced women from this issue.^39^ The first account examines the social pressures on women as they age, leading to material possessions and harsh judgements. The second narrative highlights the limited autonomy women face in arranged marriages, which can lead to drug use for coping with feelings of captivity and isolation. The third narrative explores the stigma associated with divorce.

### Barriers to treatment and recovery

‘I know stopping narcotics is beneficial for my health, but recovering is difficult. The closest centre is distant and pricey for my family. My dread of my family discovering my addiction prevented me from seeking treatment, making me feel alienated and imprisoned.’ (Participant-10, Age 33, Gender Female)

‘Drug addicts, especially women, avoid treatment in our tiny town for fear of public judgement and disgrace. Treatment centres can face public reactions that might affect the family and hinder rehabilitation. This stigma silences me, prevents me from acting and keeps me addicted.’ (Participant-1, Age 35, Gender Female)

‘My experience with a government-run treatment centre that lacks privacy causes fear and anxiety. These hospitals’ lack of anonymous, confidential treatment options poses a significant barrier, particularly in a small town where personal matters are often public. Without this assurance, I often face my addiction alone, devoid of the necessary support and professional care.’ (Participant-4, Age 29, Gender Female)

The narratives highlight the challenges faced by women dealing with drug abuse, highlighting the economic, social and institutional barriers that hinder their treatment and rehabilitation.^40^ Financial difficulties, anxiety and the stigma related to substance abuse prevent women from seeking help. This cycle of silence leads to medication adherence rather than seeking help. Government-run treatment centres lack privacy and anonymity, further deterring women from seeking help. In close-knit communities, personal challenges can become common knowledge, causing women to fear sharing their addiction struggles.

### Integrated findings

We used surveys and interviews to investigate how women use drugs and the psychological and social effects that come with the habit. A significant finding was that 70% of the participants were unemployed and earned less than 5000 PKR a month, indicating serious economic challenges. The research revealed that fluctuations in currency affected the drug use of women, as financial constraints led them to turn to substances after job loss, rising expenses or other financial difficulties.^41^ We observed that the participants were experiencing significant mental distress, as evidenced by their mean scores of 1.48, 1.41 and 1.50, all of which indicate moderate levels of depression, anxiety and stress. Regression analyses indicated that emotional support from family is associated with a decrease in drug use (*β* = −0.25, *p* = 0.02), whereas stigma is linked to an increase in drug use (*β* = 0.28, *p* = 0.01). A supportive family can potentially lower the likelihood of drug use, whereas social stigma tends to elevate the use.^42^ The societal conventions significantly influence substance use, with 63.3% of participants experiencing Social Stigma and 48.3% experiencing stress because of cultural pressure, particularly among women who resort to drug use after coerced marriages.^43^ Quantitative data indicate a negative correlation between family emotional support and drug use (*r* = −0.28, *p* = 0.01), suggesting that supportive families can reduce drug misuse. However, qualitative research reveals that some women still use drugs despite having supportive families, pointing to factors like emotional suffocation and rigid expectations as potential triggers.^44^ While 50% of participants were aware of mental health services, only 39.1% felt that they had access, with stigma and a lack of gender-sensitive facilities being significant barriers. Qualitative insights further emphasise that stigma deters women from seeking treatment, as most rehabilitation centres cater primarily to men.^45^ A regression analysis also supports the negative correlation between family emotional support and drug use (*β* = −0.25, *p* = 0.02), reinforcing the idea that strong family support can mitigate drug use. However, the analysis also indicates that some stable people take drugs owing to internalised pressures, emphasising the necessity to study psychological resilience in this setting.^35^

## Discussion

The study highlights the significant impact of poverty, unemployment and limited education on drug use among women in KP Province. With 70% of women unemployed and 75.8% earning less than 5000 PKR monthly, they resort to drug use to cope with economic stress.^15^ The lack of education severely limits employment opportunities and economic independence, increasing the risk of substance use.^11^ The study also found that women who use drugs experience significant mental health issues, with average scores indicating moderate depression, anxiety and stress.^46^ Mental health access was limited, with 39.1% of participants finding help. Many women resort to self-medication because of trauma, stress and emotional trauma, while family emotional support is lacking, further marginalising these women and reducing their likelihood of seeking professional treatment.^38^ The report also highlights the impact of gender roles and social expectations on drug use among KP women, with 63.3% reporting community Social Stigma and 48.3% feeling compelled to comply.

This research supports the idea that financial issues, mental health issues and social stigma affect women’s drug use.^47^ In North America and Europe, trauma, stress and societal factors lead to more opioid usage by women than by men.^48^ Canadian and American studies indicate that domestic violence, unemployment and psychological suffering dramatically raise women’s drug addiction risks.^49^ Economic instability and increased violence against women drive the rise in drug usage among African and Latin American women.^50^ Drug use in South Asia and the West differs, with developed nations providing better access to early intervention, addiction treatment and mental health care, while gender-sensitive rehabilitation programmes reduce stigma.^51^ In KP, strict cultural standards^52^ force many women to hide their drug use, delaying treatment and worsening their addiction. Because of a dearth of suitable rehabilitation clinics and societal stigma, KP women struggle to obtain help. Opioids, cannabis and methamphetamine are the most often utilised illegal drugs among KP women, usually acquired from friends and relatives. This study shows the necessity of gender-sensitive drug-prevention strategies.^53^

## Conclusion

The study explores the factors influencing drug use among women in KP, Pakistan. It reveals that financial difficulties, mental health issues and societal limitations make women vulnerable. These factors increase the likelihood of substance abuse, while inadequate psychological support worsens trauma and depression. The study recommends rehabilitation centres, mental health facilities, community counselling and increased education and job-training access. Government-operated support initiatives and community engagement programmes could reduce substance abuse and generate income.
